# Depressive and anxiety symptoms and associated factors among postnatal women in Enugu-North Senatorial District, South-East Nigeria: a cross-sectional study

**DOI:** 10.1186/s13690-018-0329-6

**Published:** 2019-01-10

**Authors:** Olaoluwa S. Agbaje, Joy I. Anyanwu, Prince I. C. Umoke, Tochi E. Iwuagwu, Cylia N. Iweama, Eyuche L. Ozoemena, Ijeoma R. Nnaji

**Affiliations:** 1 Department of Human Kinetics and Health Education, Faculty of Education, University of Nigeria, Nsukka, Nigeria; 2 Department of Educational Psychology, Faculty of Education, University of Nigeria, Nsukka, Nigeria

**Keywords:** Depression, Anxiety, Anxious-depressed, Mental health screening, Comorbidity, Postnatal

## Abstract

**Background:**

Postnatal depression (PND) and anxiety (PNA) among women are prevalent and impairing health problems, with adverse outcomes for mothers and their infants. This study assessed the prevalence of depression, anxiety and associated factors in a sample of postnatal women.

**Method:**

A community-based cross-sectional study was conducted on 270 postpartum women attending public health facilities in the study area. Postnatal depression was measured using the Edinburgh Postnatal Depression Scale (EPDS) and anxiety was measured using the Hospital Anxiety and Depression Scale (HADS-A). Data on maternal demographics, health characteristics, pregnancy-related characteristics, labor and birth characteristics, were collected via structured questionnaire. Binary Logistic and multinomial logistic regression analyses were carried out to identify the factors associated with depressive and anxiety symptoms in women.

**Results:**

The EPDS identified 92 women (34.6%) as possibly depressed (using a cut-off ≥13) while the HADS-A identified 89 women (33.3%) with anxiety symptoms (using a cut-off + 8). A total of 69 women were identified with symptoms of anxiety and depression (anxious-depression). The multinomial regression analysis (MLA) showed that the history of depression (AOR = 0.12, 95% (CI 0.02, 0.76), and being a mother aged 15–29 years (AOR = 10.31, 95% (CI 1.13, 94.11) had a significant effect on the development of anxiety symptoms in women. Although not significant, mother’s income level (AOR = 1.53, 95% (CI 0.72, 3.25), and being a younger mother (AOR = 1.06, 95% (CI 0.21, 5.26) were more likely to predict depressive symptoms in postnatal women. Attendance at postnatal care services in the PHCs (AOR = 0.14, 95% CI (0.04, 0.48) was significantly associated with anxious-depressed in the studied postnatal women.

**Conclusion:**

The findings of this study showed a direct association between depressive symptoms, anxiety and younger maternal age, rural residence, and low income. The higher prevalence of depressive and anxiety symptoms in this study is a call for postnatal care that is culturally sensitive, patient-centered, accessible and affordable by women, most importantly poor and rural women.

**Electronic supplementary material:**

The online version of this article (10.1186/s13690-018-0329-6) contains supplementary material, which is available to authorized users.

## Background

The birth of a newborn brings happiness, a sense of fulfillment and euphoria in women. However, many women and infants die in the first six weeks after delivery [1]. This period is also characterized by abrupt changes and stressful life events such as depression and anxiety [2, 3]. Depression and anxiety are common and debilitating complications of postpartum women [4, 5]. A meta-analysis conducted by O’Hara and Swain [3] showed that around 13% of childbearing women suffer from postnatal depression (PND). Evidence suggests that undetected or untreated depression pose a risk of adverse health outcomes for mothers, their babies, and family members [4, 6, 7]. Studies have suggested that PND has received considerable research attention while postnatal anxiety (PNA) has been relatively neglected [2, 8]. It has been established that symptoms of depression and anxiety co-occur and that this comorbidity may be an indicator of the severity of psychological distress [9].

However, evidence suggests that women also experience postnatal anxiety without depression. For example, a systematic review conducted on pre- and postnatal psychological wellbeing in African women living in Africa showed that the prevalence rate of postnatal anxiety was 14.0% [10]. Studies have pointed out the importance of distinguishing between depression and anxiety in the postnatal period in order to provide appropriate interventions that specifically target the symptoms and etiology of anxiety [4, 11, 12]. Furthermore, studies with non-postpartum populations have shown that comorbidity (simultaneous appearance of multiple illnesses) of anxiety and depression (i.e., anxious depression) manifests more severe symptoms [13]. Co-occurrence of depressive and anxiety symptoms in postnatal period is more difficult to treat than each disorder alone [14] and linked with adverse maternal and child health outcomes such as poor cognitive development and social-emotional development of the child [4, 6], increases the risk for suicide, and requires specific treatment strategies for both sets of symptoms [14, 15]. Miller et al. [2] observed that the focus on depression can result in cases of anxiety (without depression) being undetected, and untreated. The authors further averred that in cases where depression and anxiety co-occur (anxious-depression), there is a risk that treatment strategies focus on the depressive symptoms, at the preclusion of specific treatments for the symptoms of anxiety.

Few studies have reported the prevalence of depressive and anxiety symptoms in postnatal women in Nigeria [16–18]. For example, Uwakwe and Okonkwo [16] reported depression in 10% of perinatal women in Eastern Nigeria, Adewuya et al. [17] reported depression in 14.6% of postpartum women and 6.3% of general women population in Western Nigeria, while another study [18] reported depression in 22.9% of postnatal women suffered in Enugu, Eastern Nigeria. The prevalence of PND in the later studies was higher than 13% of women reported by O’Hara and Swain [4]. The higher prevalence of PND in Nigerian women may be attributed to many factors. Although maternal health service delivery in Nigeria has improved greatly in the past few decades, many challenges still undermine the quality and uptake of maternal health services by Nigerian women. For instance, poor use of maternal health services in Nigeria is a key factor contributing to high levels of maternal morbidity and mortality, as only 51.1% of women completed four or more antenatal care visits and only 36% of births took place in a health facility in 2013 [19]. Poor perceived quality of care at facilities is a critical barrier [20–22], and poor health worker attitudes contribute to a woman’s choice of using a facility or traditional provider [22].

These apparent inadequacies of health facilities, maternal health service delivery and healthcare manpower in Nigeria may suggest that many cases of PND and PNA are undetected in women or when detected may be poorly treated. The connection between depression, anxiety symptoms and maternal factors has been explored in many studies, which have, however, produced inconsistent results [8, 14, 23]. For instance, a study found 13 significant predictors of postpartum depression [23]. Some maternal factors that have been linked with PND and PNA include self-esteem, childcare stress, life stress, social support, history/previous depression, infant temperament, maternity blues, socioeconomic status, and unplanned or unwanted pregnancy, advanced maternal age, low education, low monthly income, financial difficulties, poor family support, dissatisfaction in marital life, and poor marital relationship [5, 8, 14, 23–26]. Only one Nigerian study [27] has examined the prevalence of depressive and anxiety symptoms in postpartum women.

## Methods

### Study design, setting and population

A facility-based cross-sectional study was conducted from June to September, 2017 at primary health centres (PHCs) and comprehensive/secondary health facilities in Nsukka and Obollo-Afor towns in Nsukka and Udenu Local Government Areas (LGAs), Enugu State. Private health facilities were excluded from this study because of low patronage and cost of maternal and child health services. Sometimes public health facilities provide free key MCH services or at subsidized cost. The study population comprised postpartum women accessing postnatal clinics either for immunization services or check-up. Women at different postpartum periods from 4 to 12 weeks were included in the study. This time-period was also consistent with other studies [1, 4]. The population of women in Nsukka and Udenu Local Government Areas according to the National Population Commission figures were 160,030 and 90,306 respectively [28]. In the towns, there are public and private health facilities (PHCs and comprehensive health facilities). Basic essential obstetric care is provided in the PHCs while comprehensive essential obstetric care is provided in the secondary/comprehensive hospitals. All postpartum women who attended postnatal clinics from June to September, 2017 were included in the study population. The women who had birth complications, critically ill or declined participation were excluded from the study.

### Sample size and determination procedure

The sample size in this study was estimated using a single proportion approach [29, 30]. Based on a previous study where 22.9% of the population had PND [18], we calculated a minimal sample size of 270 that would be required to give a 95% probability of measuring the prevalence of PND and PNA with 5% accuracy [29, 30]. Convenient sampling was employed to interview the women. We estimated that statistical power using the G*Power 3.1.9.2 software version developed by Faul et al. [31]. The post hoc power analysis for the logistic regression procedure was used. We used the conventional value of α = 0.05 as indicated by Hickey et al. [32]. software version developed by the large sample approximations proposed by Demidenko [33] with variance correction. The distribution of the predictor *X*, the “*X* distribution” and its parameters were specified. We specified the *X* distribution as normal, and its parameters, *X* parm μ and *X* parm σ as 0 and 1 respectively. Furthermore, we specified the number of “tail(s)” of the test as two, the *P*_*1*_ and *P*_*2*_ as 0.30 and 0.50 respectively while the odds ratio was 2.33333. The “*R*^*2*^ and other *X*” was specified as .1 and the total sample was 270. The results of statistical power analysis showed that the critical z = 1.95996, corresponding to α = β = 0.9999 (Additional file 1). The results indicate that the minimum sample size of 270 was enough for the study.

### Data collection

#### Outcome variables

PND was measured using the Edinburgh Postnatal Depression Scale-EPSD [31] while PNA was measured using the Hospital Anxiety and Depression Scale-HADS [34]. The EPDS was developed to screen postnatal women for the probability of depression [35]. The EPDS assesses depressive symptoms such as self-blame, anxiety, panic, coping, difficulty in sleeping (due to unhappiness), sadness, tearfulness and self-harm [35]. Somatic symptoms associated with the postpartum period such as extreme tiredness and loss of appetite were not included in the EPDS and which may otherwise possibly not differentiate postpartum women with or without depression. The EPDS contains questions such as “I have been able to laugh and see the funny side of things” and “I have blamed myself unnecessarily when things went wrong”. Participants are asked on a four-point Likert scale to describe their most true feelings over the past seven days using options which range from “No, not at all” (0) to “Yes, most of the time” (3). The scale has a maximum total score of 30. Many studies have reported that the EPDS has good psychometric properties, including sensitivity and specificity. A cut-off score of 12 or more on the EPDS has been recommended to detect probable depression in postpartum women [23, 35–37]. The reliability and validity of the EPDS have been well-documented [36, 38]. We used a cut-off point ≥13 on the EPDS [35]. A cut-off point ≥13 is an acceptable cut-point for identifying women at risk for major depression in clinical settings [10, 35].

The use of higher threshold is clinically justified since increased scores on the EPDS may be explained by other factors such as transient stress, and “baby blues” characterized by brief crying spells, irritability, nervousness, and poor sleep unrelated to a depressive disorder, but related to normative experiences of pregnancy [39, 40]. In the statistical analyses for associated factors in the present study the EPDS scores were dichotomized as depressed (≥ 13) or not depressed (< 13). Cronbach’s alpha for the EPDS in the present study was .73. Although EPDS contains a cluster of items (EPDS-3A, i.e., items 3, 4 and 5) to measure anxiety symptoms in postpartum women, only a few studies [36, 41] exist to validate the efficacy of EPDS-3A to detect probable anxiety symptoms in women. To complement the EPDS as a measuring tool for depression and anxiety in this study, we used the HADS-A. Numerous studies have used the HADS-A to measure anxiety symptoms in primary health care settings and general populations [34, 40, 41]. The reviews conducted by Herrmann [42] and Bjelland [43] showed that the HADS performed well in assessing the symptom severity and caseness of anxiety disorders and depression in both somatic, psychiatric and primary care patients and in the general population. The HADS is a 14-item instrument divided into two subscales for Anxiety (HADS-A) and Depression (HADS-D). We used only the Anxiety subscale (HADS-A) to measure anxiety in women. Participants were asked to tick the box that most describes how they have been feeling in the past seven days on a four-point Likert scale. The Anxiety subscale (HADS-A) has seven items. An optimal cut-off score of 8+ for both HADS-A has been recommended for use as an indicator of psychiatric morbidity according to DSM-III criteria. Bjelland [43] reported that the correlations between the two subscales varied from .40 to .74 (mean .56) while Cronbach’s alpha for HADS-A varied from .68 to .93 (mean .83). Similarly, in most studies, an optimal balance between sensitivity and specificity was achieved when caseness was defined by a score of 8 or above on both HADS-A and HADS-D [42, 44]. In the present study, we used a cut-off point + 8 on the HADS-A. In the statistical analyses for associated factors in the present study the HADS-A scores were dichotomized as anxious (+ 8) or not anxious (< 8). The Cronbach’s alpha coefficient value for the HADS-A was .70.

### Measures of associated factors

Based on the findings of previous studies, we assessed the associated factors of depressive and anxiety symptoms in postpartum women [8, 23]. In this study, we included a question about whether the mother had experience violence at home from her husband/partner in that past 12 months. Experience of violence was measured according to the WHO Domestic Violence Questionnaire [45]. Items 704 to 706 in the Questionnaire measure seven types of physical abuse, four types of psychological abuse, and three types of sexual abuse perpetrated by the husband/partner in the last 12 months. We asked women to describe their experience of domestic violence using dichotomous response option of “Yes” or “No”. If women answered “Yes” to any of the questions, it implies domestic violence experience (DVE). Items that assessed family support were adapted from the family subscale of the Multidimensional Scale of Perceived Social Support developed by Zimet et al. [46]. Items 3, 4, 8 and 11 that measure perceived family support were used in this study. The items included: “My family really tries to help me”, “I get the emotional help and support I need from my family”, “I can talk about my problems with my family” and “My family is willing to help me make decisions”. Women responded to these items using a four-point scale of “Strongly Agree” (4), “Agree” (3), “Disagree” (2) and “Strongly Disagree” (1). The MDSSS has been reported to have good psychometric properties [47]. Any mean total scale score ranging from 1.0 to 1.9 was considered low family support; a score of 2.0 to 3.0 was considered moderate family support; a score from 3.1 to 4.0 was considered high family support. The Cronbach’s alpha of the MDSSS scale was 0.75 in this study. Information about any history of depression was collected using a scale consisting of 5 questions (concerning sadness, appetite changes, lack of energy, self-blame, and concentration) constructed to measure lifetime history of major depression based on the DSM-IV criteria [26]. When a woman reported having experienced ≥3 of these symptoms simultaneously for more than 2 weeks, she was asked to specify when this had occurred: during pregnancy, after the current delivery, and/ or previously. When a woman indicated experience ≥3 of these symptoms simultaneously for more than 2 weeks, it was coded 1 (Yes) and if not coded 0 (No). Maternal demographic characteristics such as age, health facility type attended, family/household income level, parity, and birth type were obtained from the women and health facility’s birth records. These records also contained information about the present mode and time of delivery and characteristics of the infants, such as sex, twins/triplets, gestational age, birth weight, including Apgar scores. Furthermore, the women were asked about their highest completed level of education. Four categories of level of education were explored. These included no formal education (coded as 1), primary education (coded as 2), secondary education (coded as 3) and tertiary education (coded as 4). Women’s places of residence were categorized into the urban and rural areas. An urban residence was coded as 1 while rural residence was coded as 2. Facility type attended during postpartum period was classified into primary health center (coded as 1) and secondary health facility (coded as 2). Women’ age being a continuous variable was categorized into three groups. The first group was women aged 15–29 years (coded as 1), the second group comprised of women aged 30–44 years (coded as 2) and third group comprised of women aged 45 years and above (coded as 3). We created three groups of women based on the family/household monthly income using the current minimum wage in Nigeria, which is #18,000 per month since 2011. Due to the recent economic downturn, we created three income groups, namely; less than #10,000.00 per month (coded as 1), #10,000-#20,000 per month (coded as 2) and monthly income that exceeds #20,000.00 per month (coded as 3). The birth type included natural or vagina birth (coded as 1) and caesarean section (coded as 2). Parity included primiparous which was coded as 1 and multiparous was coded as 2.

### Data processing and statistical analysis

Binary logistic regression was used to identify independent effect of each independent variable on the outcome variables. The “Forced Entry” method was used to perform the binary and multivariable logistic regression analyses. Interactions were explored. Multinomial logistic regression (MLR) analysis was carried out using the variables that were significant (*p* < 0.05) at 95% CI in the bivariate analysis after checking for multicollinearity (Variance Inflation Factor [VIF] < 10). Any variable whose VIF was greater than 10 were not entered to MLR [48], Additional file 2: Tables S1, Table S2 and Table S3. For the MLR, the outcome variables included depression, anxiety, and anxious-depressed. The category of women without any symptoms (no symptoms) was used as reference category. The fitness of the binary logistic regression models was assessed using the Hosmer-Lemeshow test while the goodness of fit of the MLR was assessed using the Pearson and Deviance statistics [48]. Adjusted odds ratios (AOR) at 95% were calculated to determine the strength of the relationship between dependent and independent variables. The Wald test was used to test the statistical significance of OR. The results were considered statistically significant at *P* < 0.05. The data were entered into IBM SPSS (version 22.0 IBM Corporation, 2013) for data checking, cleaning, and recoding for analysis.

## Results

### Characteristics of the population

In this study, 270 women participated. Of the 270 respondents, 267 respondents provided a complete response to the section on demographic data, the EPDS and HADS-A. This gave a response rate of 98.9%. Participants completed the questionnaires at 4 weeks, 8 weeks and 12 weeks after childbirth. At 4 weeks, 87 mothers (32.7%) completed the questionnaires while 81 mothers (30.3%) and 99 mothers (37.1) completed the questionnaires at 8 and 12 weeks respectively (Table 1). The mean age of the women was 28.9 years (±6.2). About half of the women experienced domestic violence in the past 12 months (45.3%, *n* = 121).

**Table 1:** Socio-demographic characteristics of postpartum women

Characteristics	*n* (%)
Maternal age	
15–29 years	126 (47.2)
30–44 years	116 (43.4)
≥ 45 years	25 (9.4)
Postpartum period	
4 weeks postpartum	87 (32.6)
8 weeks postpartum	81 (30.3)
12 weeks postpartum	99 (37.1)
Child age	
1–4 weeks	64 (23.9)
5–9 weeks	80 (30.0)
≥ 10 weeks	123 (46.1)
Maternal level of education	
No formal education	55 (20.6)
Primary education	57 (21.3)
Secondary education	74 (27.7)
Tertiary education	81 (30.3)
Health facility type attended	
PHC	178 (66.7)
Secondary health facility	89 (33.3)
Place of residence	
Urban	108 (40.4)
Rural	159 (59.6)
Domestic violence experience	
Yes	121 (45.3)
No	146 (54.7)
Family support	
Poor	64 (20.0)
Moderate	80 (30.0)
High	123 (46.1).
Household/maternal income	
#10,000.00	98 (37.0)
#10,000.00 - #20,000.00	91 (34.1)
≥ #20,000.00	78 (29.2)
History of depression	
Yes	129 (48.3)
No	138 (51.7)
Birth type	
Natural birth	201 (75.3)
Caesarean section	66 (24.7)
Parity	
Primiparous	114 (42.7)
Multiparous	153 (57.3)

### Prevalence of depressive and anxiety symptoms according to the EPDS and HADS-A

The mean score on the EPDS was 11.9 (SD = ±4.1). We used a cut-off point ≥13 on the EPDS [35, 36]. Based on the cut-off point, women who scored 13 and above on the EPDS were categorized as depressed while women scoring less than 13 on the EPDS were identified as not depressed. The prevalence of PND in our sample was 34.6% on the EPDS. The prevalence of PND in our sample was higher during the 12 weeks postpartum (36.4%) (Table 2). The mean anxiety score on the HADS-A was 6.52 (SD = ±3.8). We used a cut-off score of + 8 on the HADS-A to identify groups of mothers with anxiety symptoms [38–41, 49, 50]. Based on the cut-off, women who scored + 8 on the HADS-A were categorized as anxious while women scoring less than 8 were considered not anxious. On the HADS-A, the number of women identified in our sample as anxious was 89 (33.3%). The prevalence of PNA in our sample was higher during 4 weeks postpartum on the HADS-A (41.4%) (Table 2).

**Table 2:** Prevalence of postnatal depression and anxiety according to the EPDS and HADS-A

	EPDS		HADS-A	
	Depressed ≥13	Not depressed < 13	Anxious ≥8	Not anxious < 8
	*n* (%)	*n* (%)	*n* (%)	*n* (%)
Overall	92 (34.5)	175 (65.5)	89 (33.3)	178 (66.7)
4 weeks postpartum	31 (35.6)	56 (64.4)	36 (41.4)	51 (58.6)
8 weeks postpartum	25 (30.8)	56 (69.2)	26 (32.1)	55 (67.9)
12 weeks postpartum	36 (36.4)	63 (63.6)	27 (27.3)	72 (72.7)

### Comorbidity of depression and anxiety

**Fig. 1:**
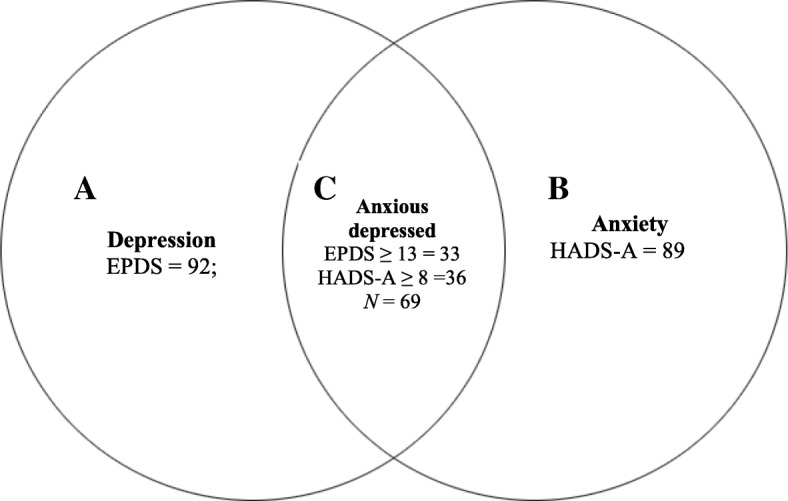
Venn diagram showing the overlapping of postpartum depression, anxiety and anxious depression among mothers. **a** – Depression (*n* = 92). **b** – Anxiety (*n* = 89). **c** – Anxious depressed (*n* = 69). Figure 1 shows that 92 and 89 women were detected by the EPDS and HADS-A with depressive and anxiety symptoms respectively. In addition, Fig. 1 shows that 69 women had co-occurrence of depressive and anxiety symptoms (anxious depressed)

In this study, we discovered another sub-group of women with both symptoms of depression and anxiety who were classified as *anxious-depression*, a termed used in an earlier study by Miller et al. [2]. To determine the sub-group of women with depressive and anxiety symptoms (anxious depressed), we used cut-off ≥13 on the EPDS and + 8 on HADS-A. Figure 1 shows the categorization of women with depression (without anxiety), anxiety (without depression) and both symptoms of anxiety and depression (anxious-depressed) in our sample. In our sample, a total of 69 women (25.8%) were *anxious-depressed* (Fig. 1).

### Factors associated with depressive and anxiety symptoms

Factors associated with the PND in women were assessed independently based on the data collected from 4 weeks to 12 weeks postpartum. We conducted bivariate analysis to identify the significant contributions of these factors for developing depressive symptoms in postnatal women. Mothers aged 45 years and above were 2 times (AOR = 2.61, 95% CI (1.04, 6.55) more likely to develop depressive symptoms as compared to mothers aged 15–29 years. Mothers who attended secondary health facilities were 2 times (AOR = 2.96, 95% CI (1.58, 5.61) more likely to develop depressive symptoms as compared to mothers who attended PHCs. Mother’s age and health facility type attended for postnatal services contributed significantly to women’s chances of developing depressive symptoms. Mother’s level of education did not contribute significantly to the model (Table 3). Interactions were examined for variables found in the second model in the binary logistic regression analysis (Additional file 2: Table S4). Among the interactions created, only postnatal clinic attendance at a secondary health facility by being a mother aged 30–44 years had significant effect on development of depressive symptoms among mothers. Hence, interaction term, attendance at a secondary health facility by being a mother aged 30–44 years, was included in the final model. This category of mothers were 16 times (AOR = 16.93, 95% CI (1.63, 175.72) more likely to develop depressive symptoms compared to mothers who attended secondary health facilities and aged 15–29 years or ≥ 45 years.

**Table 3:** Binary logistic regression identifying factors associated with PND in women

Factors	Frequency *n* (%)	AOR	95% C I	
Maternal age				
15–29 years^a^	52 (56.5)	1		
30–44 years	25 (27.2)	0.39	0.21	0.73*
≥ 45 years	15 (16.3)	2.61	1.04	6.55*
Maternal level of education				
No formal education^b^	20 (21.7)	1		
Primary education	28 (30.4)	1.60	0.71	3.62
Secondary education	20 (21.7)	0.96	0.42	2.18
Tertiary education	24 (26.1)	0.83	0.38	1.82
Health facility type attended				
PHC^c^	19 (20.7)	1		
Secondary health facility	73 (79.3)	2.96	1.58	5.61*
HFT * Maternal Age				
HFT(1) by Maternal Age(2)		16.93	1.63	175.72*
Constant		0.35		

^a^We used 15–29 years as a reference value

^b^We used no formal education as a reference value

^c^We used no PHC as a reference value

AOR = Adjusted Odds Ratio. * *p* < .05

HFT(1), denotes primary health center (PHC)

Maternal Age (2), denotes age group 30–44 years

*HFT* Health facility type

Hosmer and Lemeshow test, *p*-value = 0.612 (Model 1)

Hosmer and Lemeshow test, *p*-value = 0.908 (HFT * Maternal Age)

Bivariate analysis using logistic regression showed that being a mother below 44 years old (AOR = 0.33, 95% CI (0.18, 0.63), and having a monthly income of < #10,000.00 or ≥ #20,000.00 (AOR = 3.79, 95% CI (1.82, 7.88), were significantly associated with PNA in mothers (Table 4). Mothers with a monthly income of ≥ #20,000.00 were 3 times (AOR = 1.48, 95% CI (0.72, 3.01) more likely to develop anxiety symptoms as compared to mothers with monthly income between #10.000 - #20.000 years. Mothers with primary education were 2 times (AOR = 2.00, 95% CI (0.87, 4.62) more likely to develop anxiety symptoms compared to mothers with different levels of education. However, having a primary education was not significantly associated with PNA in mothers. DVE was not included in the analyses (VIF > 10) because of non-significance (Additional file 2: Tables S1, Table S2, and Table S3). Interactions were examined for variables found in the second model of the binary logistic regression analysis for the associated factors of PNA postnatal women (Additional file 2: Table S5). Among the interactions created, only having a secondary education by a monthly income of below #20, 000.00 and above #20, 000.00 had significant effect on development of anxiety symptoms among mothers. Hence, interaction term, having a secondary education by a monthly income of below #20,000.00 and above #20,000.00 was included in the final model (Table 4).

**Table 4:** Binary logistic regression identifying factors associated with PNA in women

Factors	Frequency *n* (%)	AOR	95% CI	
Mother’s age				
15–29 years^a^	56 (62.9)	1		
30–44 years	28 (31.5)	0.33	0.18	0.63*
≥ 45 years	5 (5.6)	0.39	0.13	1.18
Mother’s education level				
No formal education^b^	18 (20.2)	1		
Primary education	29 (32.6)	2.00	0.87	4.62
Secondary education	18 (20.2)	0.76	0.33	1.79
Tertiary education	24 (27.0)	0.85	0.39	1.88
Place of residence				
Urban^d^	29 (32.6)	1		
Rural	60 (67.4)	1.58	0.60	4.18
Maternal income^c^				
< #10,000.00^e^	27 (30.3)	1		
#10,000 - #20,000	27 (30.3)	1.48	0.72	3.01
≥ #20,000.00	35 (39.3)	3.79	1.82	7.88**
History of depression				
Yes^f^	35 (39.3)	1		
No	54 (60.7)	1.53	0.60	3.92
Educ Level * Income Level				
Educ Level(2) by Income Level(1)		0.02	0.00	0.27**
Educ level(2) by Income Level(2)		0.09	0.01	0.75*
Constant		0.28		

^a^We used 15–29 years as a reference value

^b^We used no formal education as a reference value

^c^We used no PHC as a reference value

^d^We used urban residence as a reference value

^e^We used income level < #10,000.00 as a reference value

^f^We used a history of depression as a reference value

Educ level 2 denotes the category of women with secondary education

Income level 1 denotes the category of women with a monthly income within #10,000 - #20,000

Income level 2 denotes the category of women with a monthly income of ≥ #20,000

* *p* < .05, ** *p* < .001

Hosmer and Lemeshow test, *p*-value = 0.063 (Model 1)

Hosmer and Lemeshow test, *p*-value = 0.217 (Mother’s Educ Level * Income Level)

Regarding comorbid depression and anxiety symptomatology, bivariate analysis using logistic regression showed that attendance at a secondary health facility (AOR = 3.45, 95% CI (1.39, 8.57), and having no history of depression (AOR = 3.56, 95% CI (1.21, 10.50), were significantly associated with co-occurrence of anxiety and depressive symptoms in postpartum women (Table 5). Mothers who attended a secondary health facility were 3 times more likely to develop anxiety and depressive symptoms concurrently as compared to mothers who attended a PHC. In addition, mothers with no history of depression were 3 times more likely to develop anxiety and depressive symptoms concomitantly as compared to mothers with history of depression. Although not significant, mothers aged 45 years and above (AOR = 6.68, 95% CI (0.92, 48.44), and mothers with secondary education (AOR = 6.47, 95% CI (0.81, 51.54), were 6 times more likely to have comorbidity of anxiety and depressive symptoms compared to mothers aged 15–29 years and those with no formal education respectively. Mothers with a monthly income within #10,000-#20,000 (AOR = 3.97, 95% CI (0.64, 24.74) were 3 times more likely to develop the comorbid conditions as compared to mothers with a monthly income below #10,000. However, the result was not significant. As shown in Table 5, being aged 30–44 years is a protective factor against the development of comorbidity of depressive and anxiety symptoms in women, reducing the risk by 58%. Having a rural residence is also a protective factor against the development of comorbid depressive and anxiety symptoms. Rural postpartum women are 54% less likely to develop the comorbid conditions than urban women. Interactions were examined for variables found in the second model of the binary logistic regression analysis for the factors associated with comorbidity of anxiety and depressive symptoms in women (Additional file 2: Table S6). Among the interactions created, only having a secondary education by a monthly income within #10,000 - #20, 000.00 had significant effect on having comorbidity of anxiety and depressive symptoms in women. Hence, interaction term, having a secondary education by a monthly income within #10,000 - #20, 000.00 was included in the model (Table 5).

**Table 5:** Binary logistic regression identifying factors associated with comorbidity of anxiety and depressive symptoms in women

Factors	Frequency *n* (%)	AOR	95% CI	
Mother’s age				
15–29 years^a^	41 (59.4)	1		
30–44 years	19 (27.5)	0.42	0.08	2.21
≥ 45 years	9 (13.1)	6.68	0.92	48.44
Mother’s education level				
No formal education^b^	13 (18.8)	1		
Primary education	24 (34.8)	2.52	0.42	15.28
Secondary education	13 (18.8)	6.47	0.81	51.54
Tertiary education	19 (27.5)	1.52	0.19	11.91
Health facility type attended				
PHC^c^	14 (20.3)	1		
Secondary health facility	55 (79.7)	3.45	1.39	8.57**
Place of residence				
Urban^d^	25 (36.2)	1		
Rural	44 (63.8)	0.46	0.15	1.40
Maternal income				
< #10,000.00^e^	27 (39.1)	1		
#10,000 - #20,000	21 (30.4)	3.97	0.64	24.74
≥ #20,000.00	21 (30.4)	0.92	0.14	6.26
History of depression				
Yes^f^	31 (44.9)	1		
No	38 (55.1)	3.56	1.21	10.50*
Mother’s Educ Level * Income Level				
Sec. Education by #10,000 - #20,000		0.06	.01	0.77*
Constant		0.10		

*Note*. No symptoms (reference category)

^a^We used 15–29 years as a reference value

^b^We used no formal education as a reference value

^c^We used PHC as a reference value

^d^We used urban residence as a reference value

^e^We used income level < #10,000.00 as a reference value

^f^We used a history of depression as a reference value

Sec. denotes secondary

* *p* < .05, ** *p* < .001

Hosmer and Lemeshow test, p-value = 0.239 (Model 1)

Hosmer and Lemeshow test, p-value = 0.690 (Mother’s Educ Level * Income Level)

A Multinomial Logistic Regression (MLR) was used to analyze associated factors for four categories of postnatal women such as women without depressive and anxiety symptoms or their comorbidity (no symptoms), women with depressive symptoms only, women with anxiety symptoms only and women with both anxiety and depressive symptoms (anxious-depressed). The reference category for the outcome variable was “no symptoms”; each of the other three categories was compared to this reference group. The analysis was focused on the relationship between history of depression, income level, parity and depressive and anxiety symptoms (four categories) while controlling for maternal age, level of education, health facility type and place of residence. The first column in Table 5 has the outcome of “anxiety” compared to “no symptoms”. The results indicate that a history of depression (AOR = 0.12, 95% (CI 0.02, 0.76) had a significant effect on the development of anxiety symptoms in women. However, the income level of women (AOR = 1.82, 95% (CI 0.85, 3.87) is more likely to increase the likelihood of anxiety symptoms. Being a younger mother was significantly associated with anxiety symptoms. Women aged 15–29 years were 10 times (AOR = 10.31, 95% (CI 1.13, 94.11) more likely to develop anxiety symptoms than women aged ≥45 years while women aged 30–44 years are 7 times (AOR = 7.25, 95% (CI 0.81, 64.65) more likely to develop anxiety symptoms than women aged ≥45 years. Attendance at postnatal care services in PHCs (AOR = 0.32, 95% CI (0.10, 1.00) was significantly associated with anxiety symptoms. A mother’s level of education and place of residence were not significant predictors of anxiety symptoms.

The second column in Table 6 has the outcome of “depressive symptoms” compared to “no symptoms” (reference category). Although not significant, mother’s income level (AOR = 1.53, 95% CI (0.72, 3.25), and being a younger mother (AOR = 1.06, 95% CI (0.21, 5.26) were more likely to predict depressive symptoms in the studied postnatal women. A mother’s level of education, health facility type attended and place of residence were not significantly associated with depressive symptoms.

**Table 6:** Multinomial logistic regression model identifying factors associated with comorbid anxiety and depressive symptomology in postnatal women

	Anxiety			Depression			Anxious-depressed		
Variable	AOR	(95% CI)		AOR	(95% CI)		AOR	(95% CI)	
History of Depression	0.12	0.02	0.76*	0.22	0.04	1.21	0.52	0.09	3.18
Income Level	1.82	0.85	3.87	1.53	0.72	3.25	1.90	0.86	4.19
Parity	0.53	0.17	1.61	0.75	0.25	2.25	1.23	0.38	3.96
Mother’s Age									
15–29 years	10.31	1.13	94.11*	1.06	0.21	5.26	2.09	0.37	11.99
30–44 years	7.25	0.81	64.65	0.76	0.16	3.72	0.55	0.09	3.26
≥ 45 years	1								
Mother’s Level of education									
No formal education	2.24	0.57	8.85	1.84	0.48	7.01	2.61	0.62	10.96
Primary education	2.28	0.49	10.57	1.50	0.33	6.87	4.63	0.98	21.74
Secondary education	2.52	0.68	9.31	1.77	0.50	6.34	2.66	0.66	10.64
Tertiary education	1								
Health Facility Type									
PHC	0.32	0.10	1.00*	0.34	0.11	1.05	0.14	0.04	0.48*
Secondary HF	1								
Place of Residence									
Urban	0.21	0.03	1.68	0.90	0.13	6.00	1.09	0.15	8.13
Rural	1								
Mother’s Educ*HFT									
Sec. Educ* Secondary HFT	9.67	1.04	89.59*	3.39	0.36	32.19	5.426	0.57	51.76
Primary Educ* PHCs	8.09	0.83	79.26	4.26	0.44	41.48	10.22	1.04	100.14*
Income Level*Hist. of Dep.									
< #10,000* Hist. of Dep.	0.39	0.12	1.22	0.24	0.09	.75*	0.33	0.10	1.09
#10,000- #20,000*Hist. of Dep.	0.48	0.16	1.43	0.28	0.10	.81*	0.54	0.18	1.65
≥ #20,000*Hist. of Dep.	0.55	0.17	1.83	0.30	0.09	.98*	0.48	0.14	1.67

*Note*. Reference category: No symptoms (*n* = 23). *AOR* Adjusted Odds Ratio, *95% CI* Confidence Interval. * *p* < .05. Pearson = .03. Deviance = .41. *Sec. Educ*. secondary education. *Hist. of Dep*. History of depression. Pearson = .069. Deviance = .234 (Model for Interaction Terms)

The third column in Table 6 has the outcome of comorbidity of anxiety and depressive symptoms, i.e., anxious-depressed, compared to women with no symptoms. Attendance at postnatal care services in PHCs (AOR = 0.14, 95% CI (0.04, 0.48) was significantly associated with anxious-depressed. Although not significant, statistical analysis shows that a mother’s income level (AOR = 1.90, 95% (CI 0.86, 4.19) and parity (AOR = 1.23, 95% CI (0.38, 3.96) have the potential to increase the likelihood of anxious-depressed. Although not significant, women aged 15–29 years were 2 times (AOR = 2.09, 95% CI (0.37, 11.99) more likely to develop comorbidity of anxiety and depressive symptoms compared to older women. Similarly, women with primary education were 4 times (AOR = 4.63, 95% CI (0.98, 21.74) more likely to develop comorbidity of anxiety and depressive symptoms as compared with women with tertiary education. Also, mothers with NFE (AOR = 2.61, 95% CI (0.62, 10.96) and secondary education (AOR = 2.66, 95% CI (0.66, 10.64) were 2 times more likely to develop comorbidity of anxiety and depressive symptoms compared with women with tertiary education. Among the interactions created, having a secondary education by attendance at a secondary health facility had significant effect on occurrence of depressive symptoms in women (AOR = 9.67, 95% CI (1.04, 89.59). Having a primary education by attendance at primary health centers (PHCs) was associated with development of comorbid anxiety and depressive symptomatology in women (AOR = 10.22, 95% CI (1.04, 100.14). However, in this model, having a monthly income of < #10,000 or #10,000 - #20,000 or ≥ #20,000 and a history of depression are protective factors against development of depressive symptoms in women. According to Table 6, women with a monthly income of < #10,000 and a history of depression, women with a monthly income of #10,000 - #20,000 and a history of depression, women with a monthly income of > #20,000 and a history of depression are 76, 72 and 62% less likely to develop depressive symptoms compared to women with different levels of income and no history of depression respectively (Additional file 2: Table S7).

## Discussion

This study aimed to determine depressive symptoms, anxiety and associated factors among postpartum women. In this study, the prevalence of PND in our sample was relatively high compared to previous findings in Nigeria. The prevalence of PND was higher than the previously reported prevalence of 13% in a meta-analysis [4] 10% in Eastern Nigeria [16], 14.6% in western Nigeria [17], and 22.9% in Enugu metropolis [18]. The similar findings consistently suggest that women suffer from depression during the postpartum period. The finding also corroborates the evidence that PND is very contextual and its prevalence varies between different cultures and communities. The higher prevalence of PND in our study may be attributed to the recent economic recession and challenges facing women, especially in the rural communities. Another reason for the higher prevalence of depressive symptoms in our sample may be attributed to the influx of women with traumatic experiences of communal or herdsmen-and-farmers-related conflicts who registered for ANC services in the study area. The finding suggests the need for protection of women from exposure to conflicts or traumatic experiences, context-specific mental health interventions (e.g., cognitive behavior therapy) for postpartum women to promote mental wellbeing, prevent maternal and child adverse health outcomes. Our study further shows that the prevalence of PND was higher at the 12 weeks postpartum. This probably indicates women’s prolonged exposure to stressful events during this period. Consistent with previous findings, the 12-week postpartum has been reported as one of the peak periods of PND [4].

The point prevalence of anxiety symptoms in our sample was high. The prevalence of PNA was higher than the previously reported prevalence of 13% in Australian postpartum women [2], 12.7% in Australian women [3], and 13.1% in Arab postpartum women [8]. This finding indicates that a considerable proportion of women suffers from anxiety during the postnatal period. The disparities in the prevalence of anxiety symptoms in postpartum women in the previous studies and present study may be due to variations in cultural settings, use of different screening tools for psychiatric morbidity and cut-off points in the identification of women with anxiety symptoms. For instance, Bener et al. [8] investigated the prevalence of psychiatric morbidity in Arab women using DASS-21 with a cut-off ≥8 to identify women with anxiety symptoms. In contrast, we used HADS-A with a cut-off + 8 to identify women with anxiety symptoms. Regardless of the screening tools and cut-off used, identification of women with psychiatric morbidity in postpartum period requires proper and quality mental health intervention. The higher prevalence of anxiety symptoms in our sample may be due to the fear of newborn, displacement or detachment of some postpartum women from their support groups as a result of communal violence and the changing family structure in many Nigerian societies from extended to isolated nuclear families [17], which deprives many young women of support from their family members especially older experienced women. This situation might have heightened anxiety levels in the women. In addition, our study shows that the point prevalence of anxiety symptoms was at its peak during the 4 weeks postpartum. This finding is consistent with the previous study who reported that 30% of new postpartum women were emotionally distressed [51]. The higher prevalence of anxiety symptoms in this study is a call for postnatal care that is culturally sensitive, patient-centered, accessible and affordable by women, most importantly rural women. Since evidence suggests that poorer, less educated, and rural women have lower coverage of postnatal care [1]. Furthermore, fragile family ties which have characterized modern family structures should be strengthened to galvanize support for postpartum women. Besides, stakeholders in the integrated maternal, newborn and child health (IMNCH) and the government at all levels should strengthen the health systems for improved quality of care for mothers and babies.

Interestingly in this study, a considerable proportion of women were *anxious-depressed*. A lower prevalence of 7% was found in a study [2] that used DASS-21 to assess comorbidity of anxiety and depressive symptoms in postpartum women. The higher point prevalence of comorbidity of anxiety and depressive symptoms in our sample may be attributed to a myriad of factors such as prevailing economic situation, poor quality of maternal health services, mistreatment and disrespectful care by health workers at the health facility, exposure to traumatic experiences such as communal violence [19–22]. Evidence showed that women in low-and-middle-income countries (LMICs) such as Nigeria lack access to quality and adequate postnatal care coverage [1, 52, 53].

In our study, the following factors were associated with the occurrence of depressive symptoms in women: maternal age, and health facility type. No significant association was found between depressive symptoms and a history of PND symptoms. However, in this study, about half of the women had a history of depression. This finding contradicts existing evidence which shows that a personal history of depression is a strong risk factor for developing depressive symptoms during pregnancy and postnatal period [2–5, 8, 14, 23, 54]. The non-existence of association between a personal history of depression and PND in our sample may be due to some contextual factors such as cultural influence, study setting and method adopted. For instance, some cultural factors such as domestic violence (e.g., wife beating) and male child preference might have heightened anxiety symptoms in women especially women who with female babies whose husbands or family members expected to have male children. Such women received little or no family support and care from their husbands and family members. This finding contradicts the finding of a previous study which reported that women with a personal history of a major depressive episode are known to be at a 30% higher risk of developing PND [23].

In the bivariate analysis, mothers aged 45 years and above were 2 times more likely to develop depressive symptoms as compared to mothers aged 15–29 years and attendance at a secondary health facility was significantly associated with PND in women. Maternal level of education was not significant. The finding is inconsistent with the finding of a study [8] which reported more depressive symptoms in women under 30 years old. The association between health facility attended during pregnancy and postnatal period and development of PND probably be due to maltreatment of women by health workers during childbirth [55]. This finding is consistent with studies that have identified associations between sociodemographic factors and PND in women [23, 52, 56]. Evidence showed that being a younger mother is a risk factor for the development of PND [8] and attendance at a health facility characterized by the maltreatment of women by health workers during childbirth is a risk factor for adverse maternal outcomes [55]. Therefore, our interaction terms result showed that being a mother aged 30–44 years and attendance at a secondary health facility characterized by maltreatment of women by health workers during childbirth or postnatal period had a profound significant effect on the development of depressive symptoms among mothers. This finding advocates the importance of providing age-specific mental health interventions for women during prenatal and postnatal periods in Nigeria. This may reduce the prevalence of PND among women especially younger women. Furthermore, implementation and enforcement of valid measures targeted at preventing mistreatment/abuse of women during the childbirth and postnatal period by health workers should be encouraged at all the levels of health facility in Nigeria. This will reduce to very large extent cases of maternal mistreatment and in turn prevent or reduce significantly associated adverse outcomes.

The logistic regression analysis showed that being a mother under 44 years old, and having monthly income higher than #20,000.00 were significantly associated with PNA. Mothers with monthly income ≥ #20,000.00 were 3 times likely to develop anxiety symptoms as compared to mothers with monthly income < #10,000.00. Many studies have reported associations between low socioeconomic factors, anxiety and depressive symptoms in postnatal women [5, 8, 23, 52, 57, 58]. For instance, Segre et al. [58] reported that women who earned the higher income ($70,000 annually) were at 4 times lower risk of developing PND than with women with low incomes ($10,000 annually). In Nigeria, women with #10,000 or #20,000 monthly income, earn #120,000 or #240,000 annually respectively. This is equal to $333 or $667 annually respectively (current exchange rate = #360/$1). Consequently, women with low incomes experience hardship in raising their infants in Nigeria. Poor or limited financial resources might have increased anxiety symptoms in postnatal women. The gloomy economic reality compounded this situation and portends danger for women with low incomes who cannot afford quality postnatal care. This poses serious threats to maternal and child survival in Nigeria. Although not significant, mothers with primary education were 2 times more likely to develop anxiety symptoms compared to mothers with NFE. This finding was also observed in previous studies that low level of education is a strong risk factor for PND in women [58–60].

In this study, we found a significant association between attendance at postnatal care clinics in a secondary health facility and co-occurrence of anxiety and depressive symptoms in our sample.

Studies have reported significant association between low socioeconomic factors and psychiatric conditions in women population [23, 52, 57, 58], however, there seems to be a dearth of studies on relationship between the health facility type attended during perinatal or postnatal period and comorbid depressive and anxiety symptomatology in Nigerian postpartum women. This association may be attributed to maltreatment of women by health workers during childbirth and barriers to quality maternal care at the secondary health facilities especially in Nigerian rural communities [20–22, 55]. More research is needed to examine their relationship to one another in Nigerian context. Such research could substantially improve our understanding of this relationship with the aim of improving the conditions of maternal care services across the levels of health facilities in Nigeria. Having no history of depression was significantly associated with the comorbidity of anxiety and depressive symptoms. This finding defies existing evidence in our sample. However, the observed association may be due to the interplay of other factors not considered in this study. This finding disagrees with the previous studies that reported an association between previous psychiatric history and the risk of developing depressive symptoms during pregnancy and postnatal period [2–5, 8, 14, 23, 54]. Our interaction terms result showed that having a secondary education by a monthly income within #10,000 - #20,000 was significantly associated with the comorbid depressive and anxiety symptomatology in postpartum women. This is consistent with previous studies suggesting that low socioeconomic factors constitute strong risk factors for psychiatric conditions in women [23, 55, 57–60]. Given the attendant adverse effects of comorbid postpartum depressive and anxiety symptoms on maternal health and infant development, health care workers should intensity their efforts at identifying this sub-category of women, mitigating the risk factors and providing prompt care, referral, and/or treatment at subsidized or affordable costs.

In the MLR, attendance at postnatal care services in PHCs (AOR = 0.32, *p* = .05) was significantly associated with anxiety symptoms. This finding is consistent with other studies [8, 23, 52] who reported an association between mothers’ sociodemographic factors and anxiety disorders. A mother’s level of education and place of residence were not significantly associated with anxiety symptoms. This finding is inconsistent with other studies [8, 23, 52] who reported an association between mothers’ sociodemographic factors and anxiety disorders. Although not significant, mother’s income level, and being a younger mother were more likely to predict depressive symptoms in the studied postnatal women. This finding is concordance with other studies that reported the association between low income level, younger mother’s age and depressive symptoms [3, 5, 8, 52]. We found no association between a mother’s level of education, health facility type attended, place of residence and depressive symptoms. This finding is inconsistent with other studies [5, 8, 23, 52] who reported an association between mothers’ sociodemographic factors and depressive symptoms. However, this finding is consistent with Fiala et al. [54], who reported no significant association between PND and maternal education.

We found a significant association between attendance at postnatal care services in PHCs, and co-occurrence of anxiety and depressive symptoms in our sample. Although not significant, a mother’s income level and parity have the potential to increase the likelihood of anxious-depressed. In the same way, women aged 15–29 years were 2 times more likely to develop comorbidity of anxiety and depressive symptoms compared to older women. Similarly, women with primary education were 4 times more likely to develop comorbidity of anxiety and depressive symptoms as compared with women with tertiary education. Also, mothers with NFE and secondary education were 2 times more likely to develop comorbidity of anxiety and depressive symptoms compared with women with tertiary education. These findings are consistent with other studies [23, 48, 57–59]. This finding further corroborates the assertion of Miller et al. [2] that this sub-group of women should be identified and given appropriate interventions. However, such intervention should be context specific, culturally-sensitive, patient-centered, accessible and affordable. Provision of incentives such as free postnatal care, free transport, voucher schemes, elimination of mistreatment and disrespect [1, 61] have been identified to be effective in facilitating equity of access to quality postnatal care [1].

In the model for interaction terms, we found a significant association between having a secondary education by attendance at a secondary health facility and the development of depressive symptoms in women. We also found a significant association between having a primary education by attendance at primary health centers (PHCs) and the development of comorbid anxiety and depressive symptomatology in women. This finding is consistent with previous studies who reported a significant association between psychiatry disorders and low socioeconomic status in women [23, 57–60]. This association may be attributed to low literacy among rural women, mistreatment or abuse of women by health workers during childbirth and barriers to quality maternal care at both the PHCs and secondary health facilities in Nigeria [20–22, 55]. More research is needed to examine their relationship to one another in the Nigerian context. Such research could substantially improve our understanding of this relationship with the aim of improving the literacy level of women, fostering positive attitudes towards women by healthcare workers, scaling up conditions of maternal care services across the levels of health facilities in Nigeria. In addition, having a monthly income of < #10,000 or #10,000 - #20,000 or ≥ #20,000 and a history of depression are protective factors against the development of depressive symptoms in women. This finding is inconsistent with previous studies [2–5, 8, 14, 23, 62]. The plausible explanation for this finding could be that women with previous history of depression regardless of income level might have developed resilience to the onset of new depressive symptoms, especially if properly treated using effective interventions [62]. Furthermore, in Nigerian cultural context, older women in the family and members of women’s associations either in the religious or traditional settings provide support for postnatal women with psychiatric disorders. For instance, such measures may include offering counseling and prayer sessions, provision of material and financial incentives for the affected women. These measures strengthen the capacity of women to cope effectively with psychiatric disorders and enhance their resilience traits. This could explain the null association between income, history of depression and the development of depressive symptoms in our sample.

## Strength and limitation

The conclusion of this study was based on primary data via rigorous descriptive and analytic data analysis. Furthermore, the majority of our findings are consistent with those available in the literature. This is a correlational study and only provides information on significant associations between sociodemographic factors and depressive and anxiety symptoms in women, and it cannot be used to infer causality of these events, which would require clinical trials and longitudinal studies. The higher prevalence of depressive and anxiety symptoms and their comorbidity in the studied postnatal women could be attributed to other variables not examined in this study. For instance, variables such as unintentional pregnancy, living alone/single mother, feelings of sadness about being pregnant, prenatal depression and anxiety, breastfeeding, occupation, parental consanguinity and number of people living in the home have been identified as risk factors for both postnatal depressive and anxiety symptoms [5, 8, 23, 54]. Another limitation is that the study used both the EPDS and HADS-A scales to detect depressive and anxiety symptoms in women. The EPDS and HADS-A are suboptimal tools for detecting clinically significant depressive syndrome and they cannot replace a systematic Clinical Interview Schedule. The EPDS and HADS contain several items that are non-specific for depression. For instance, both tools can be used to detect anxiety symptoms in clinical and non-clinical settings [31–33, 37–41, 49, 50]. Also, there is a general lack of agreement on the use of specific cut-off for depression detection on the EPDS. However, there seems to be a consensus on the use of cut-off point + 8 on HADS-A for identifying women at risk for anxiety symptoms. The original studies recommend using an optimal cut-off of 10 points or higher on the EPDS [35] and an optimal cut-off point + 8 on the HADS-A based on ICD-9 and DSM-III [38, 45, 46], which are still adopted by many researchers. Some authors recommended that an EPDS optimal cut-off of 12 points or higher is an accepted cut-off for recognizing patients at risk of PND [35, 40]. Some authors consider a threshold of 13 points [40, 63] while other studies recommended a lower cut-off of nine points [2]. In this study, we used the EPDS with an optimal cut-off point of ≥13 [40, 63] and an optimal cut-off point of + 8 on the HADS-A [42, 43]. The Hosmer-Lemeshow (HL) test *p*-values for the models on associated factors of anxiety symptoms in this study appear to be low. However, the p-values (factors associated with PNA, *p* = .063; PNA interaction terms, *p* = .217) are greater than 0.05, which are indicative of good fit. However, the HL test has some limitations. For instance, HL test does not account over-fitting of the model and have a tendency to have low power. In addition, HL test offers very little direction on how to select the number of subgroups. The selection of the groups (g) is subject to the researcher’s discretion. Small values for g give the HL test less chance to detect mis-specifications. Larger values imply that the number of items in each subgroup may be too small to find differences between observed and expected values. Thus, in many cases, the selection for g is often unclear and may be arbitrary. Also, we used a small sample in this study, and evidence suggests that in a small sample, a high *p*-value from the HL test may simply be due to the test having lower power to detect mis-specification, rather than being indicative of good fit [64]. We can reasonably conclude that our models demonstrated a good fit for the data used in this study. Another limitation of this study is data collection bias or interviewer bias. Interviewers were briefed on data collection to assure consistency within and between observers. This approach may not be enough to avoid interviewer bias. However, to minimize the interviewer bias, we used well validated scales for data collection. Also, interviewers are experts who have acquired quality training on data collection process. Thus, we believe these measures can minimize or mitigate the inter-observer variability when many personnel are involved in data collection and entry [65]. Also, in this study, data were collected retrospectively, this might introduce recall bias. To mitigate the recall bias effect, women who gave a live birth in 2017 only were recruited for the study and well validated scales were used for data collection. Our analyses were adjusted for PND and PNA potential predictors such as domestic violence, birth type, family support and parity, however, there were no relationships between the factors and occurrence of PND and PND in the postnatal women. Besides, the self-reported nature of the EPDS, HADS-A and WHO’s DVE questionnaire permit response bias. This may be of particular concern for sensitive items especially in the EPDS, and WHO’s DVE questionnaire, which may lead to underreporting and therefore, may likely bias the estimated associations to null. The small sample size is another limitation of this study. Single population proportion formula was used to derive optimum sample size for this study. However, sample size calculated through single population formula was considered adequate enough to identify associated factors of depressive and anxiety symptoms in women. In this study we conducted a post hoc power analysis and obtained a power (1-β err prob) of 0.9999 which showed that the maximum permissible sample size was used for the study. Thus, we concluded that the power is sufficient for the study. Although, Schulz and Grimes [66] argued about the irrelevance of post hoc power calculations, perhaps, not calculating the statistical power at all seems to be undesirable. Our study sample consisted of only the postpartum women visiting the health facilities for postnatal care services. The sample does not include postpartum women using alternative postnatal care services provided by traditional births attendants (TBAs) and traditional healers, thus limiting the generalizability of the findings. Future studies should consider using a larger, randomized and more representative sample size.

## Conclusion

This study assessed depressive and anxiety symptoms and associated factors among postpartum women. The study results provided a basic understanding of associated factors of depressive and anxiety by symptoms in Nigerian postpartum women. The findings of this study showed a higher prevalence of depressive and anxiety symptoms in women compared to previous studies. The proportion of women with comorbidity of depressive and anxiety symptoms was also higher compared to the previous finding. There were direct associations between PND, PNA and maternal age, level of education, health facility type attended, rural residence, household or maternal income, and history of depression. Thus, the results suggested context specific, culturally-sensitive, patient-centered, accessible and affordable postnatal care interventions such as routine mental health screening during antenatal and postnatal periods become necessary to foster maternal and child survival in Nigeria.

## Additional files

Additional file 1: Appendix 1-Supplemental Tables. (DOCX 78 kb)

Additional file 2: Statistical Power Analysis Results. (DOCX 69 kb)

## Abbreviations

AOR: Adjusted odds ratio
CHCs: Comprehensive Health Centres
CI: Confidence interval
DVE: Domestic violence experience
EPDS: Edinburgh Postnatal Depression Scale
HADS: Hospital Anxiety and Depression Scale
LGA: Local Government Area
NFE: No Formal Education
PHCs: Primary health Centres
VIF: Variance Inflation Factor
