# Supplementary material for: Depressive and anxiety symptoms and associated factors among postnatal women in Enugu-North Senatorial District, South-East Nigeria: a cross-sectional study
Source: Arch Public Health. 2019 Jan 10;77:1. doi: 10.1186/s13690-018-0329-6 (PMC6327551; doi:10.1186/s13690-018-0329-6)
Supplement: Supplementary file 1 — Appendix 1-Supplemental Tables. (DOCX 78 kb) [file 13690_2018_329_MOESM1_ESM.docx]

[1] *-- Thursday, November 15, 2018 -- 19:35:45*

**z tests -** Logistic regression

**Options:** Large sample z-Test, Signorini (1991)

**Analysis:** Post hoc: Compute achieved power

**Input:** Tail(s) = Two

Odds ratio = 2.3333333

Pr(Y=1|X=1) H0 = 0.3

α err prob = 0.05

Total sample size = 270

R² other X = .1

X distribution = Normal

X parm μ = 0

X parm σ = 1

**Output:** Critical z = 1.9599640

Power (1-β err prob) = 0.9999951

Central and non-central distributions

X-Y plot for range for a range of values
