# Supplementary material for: Depressive and anxiety symptoms and associated factors among postnatal women in Enugu-North Senatorial District, South-East Nigeria: a cross-sectional study
Source: Arch Public Health. 2019 Jan 10;77:1. doi: 10.1186/s13690-018-0329-6 (PMC6327551; doi:10.1186/s13690-018-0329-6)
Supplement: Supplementary file 2 — Statistical Power Analysis Results. (DOCX 69 kb) [file 13690_2018_329_MOESM2_ESM.docx]

**Additional file 2**

**Supplemental Table 1.** Exploration of multicollinearity in the model on depression

| **Coefficients^a^** | | | | | | | | | |
| --- | --- | --- | --- | --- | --- | --- | --- | --- | --- |
| Model | Unstandardized  Coefficients | | Unstandardized  Coefficients | t | Sig. | 95.0% C.I for B | | Collinearity  Statistics | |
|  | B | S.E. | Beta |  |  | Lower bound | Upper bound | Tolerance | VIF |
| (Constant) | 10.272 | 1.662 |  | 6.181 | .000 | 6.999 | 13.545 |  |  |
| Age | -.288 | .379 | -.046 | -.759 | .449 | -1.035 | .459 | .911 | 1.098 |
| Level of Education | -.563 | .215 | -.155 | -2.621 | .009 | -.986 | -.140 | .971 | 1.030 |
| Health Facility Type | 1.990 | .549 | .232 | 3.623 | .000 | .908 | 3.072 | .826 | 1.210 |
| Place of Residence | 1.548 | .935 | .188 | 1.656 | .099 | -.293 | 3.390 | .263 | 3.800 |
| DVE | -4.209 | 1.607 | -.519 | -2.620 | .009 | -7.373 | -1.045 | .087 | 11.548 |
| Family Support | .425 | .496 | .085 | .856 | .393 | -.552 | 1.401 | .346 | 2.892 |
| Monthly Income level | .243 | .402 | .049 | .606 | .545 | -.548 | 1.034 | .525 | 1.903 |
| History of Depression | 1.898 | 1.436 | .235 | 1.322 | .187 | -.930 | 4.726 | .108 | 9.294 |
| Type of Birth | .252 | .618 | .027 | .407 | .684 | -.966 | 1.469 | .779 | 1.284 |
| # Previous Preg. | .659 | .527 | .081 | 1.250 | .212 | -.379 | 1.698 | .814 | 1.228 |
| Postpartum Period | -.631 | .395 | -.130 | -1.597 | .111 | -1.409 | .147 | .511 | 1.956 |

a. Dependent variable: postnatal depression (EPDS Score ≥ 13)

DVE, Domestic violence experience. VIF, variance inflation factor. S.E. = Standard Error. C.I. = Confidence interval

**Supplemental Table 2**. Exploration of multicollinearity in the model on anxiety

| **Coefficients^a^** | | | | | | | | | |
| --- | --- | --- | --- | --- | --- | --- | --- | --- | --- |
| Model | Unstandardized  Coefficients | | Unstandardized  Coefficients | t | Sig. | 95.0% confidence interval for B | | Collinearity  Statistics | |
|  | B | Std. Error | Beta |  |  | Lower bound | Upper bound | Tolerance | VIF |
| (Constant) | 5.516 | 1.507 | -.234 | 3.662 | .000 | 2.550 | 8.483 |  |  |
| Age | -1.357 | .344 | -.130 | -3.945 | .000 | -2.034 | -.680 | .911 | 1.098 |
| Level of Education | -.440 | .195 | .120 | -2.259 | .025 | -.823 | -.056 | .971 | 1.030 |
| Health Facility Type | .958 | .498 | .197 | 1.924 | .055 | -.023 | 1.939 | .826 | 1.210 |
| Place of Residence | 1.512 | .847 | -.090 | 1.784 | .076 | -.157 | 3.181 | .263 | 3.800 |
| DVE | -.685 | 1.457 | .160 | -.470 | .639 | -3.553 | 2.184 | .087 | 11.548 |
| Family Support | .745 | .450 | .158 | 1.658 | .099 | -.140 | 1.631 | .346 | 2.892 |
| Monthly Income level | .737 | .364 | .108 | 2.024 | .044 | .020 | 1.454 | .525 | 1.903 |
| History of Depression | .816 | 1.302 | -.086 | .627 | .531 | -1.747 | 3.380 | .108 | 9.294 |
| Type of Birth | -.748 | .560 | .036 | -1.334 | .183 | -1.851 | .356 | .779 | 1.284 |
| # Previous Preg. | .278 | .478 | -.257 | .581 | .562 | -.663 | 1.219 | .814 | 1.228 |
| Postpartum Period | -1.164 | .358 | -.234 | -3.250 | .001 | -1.869 | -.459 | .511 | 1.956 |

a. Dependent variable: postnatal anxiety (HADS-A Score +8)

*Note*. DVE, Domestic violence experience. VIF, variance inflation factor

| **Supplemental Table 3**. Exploration of multicollinearity in the model on comorbidity of anxiety and depression | | | | | | | | | | | | |
| --- | --- | --- | --- | --- | --- | --- | --- | --- | --- | --- | --- | --- |
| **Coefficients^a^** | | | | | | | | | | | | |
| Model | | Unstandardized Coefficients | | Standardized Coefficients | t | Sig. | 95.0% C.I. for B | | Collinearity  Statistics | | | |
|  |  | B | Std. Error | Beta |  |  | Lower Bound | Upper Bound | Tolerance | | | VIF |
|  | (Constant) | .163 | .188 |  | .869 | .386 | -.207 | .533 |  |  |  |  |
|  | Age | -.055 | .043 | -.082 | -1.288 | .199 | -.140 | .029 | .911 |  |  | 1.098 |
|  | Level of Education | -.024 | .024 | -.062 | -1.003 | .317 | -.072 | .023 | .971 |  |  | 1.030 |
|  | HFT | .156 | .062 | .168 | 2.508 | .013 | .033 | .278 | .826 |  |  | 1.210 |
|  | Place of Residence | .067 | .106 | .075 | .636 | .525 | -.141 | .275 | .263 |  |  | 3.800 |
|  | DVE | -.370 | .181 | -.420 | -2.037 | .043 | -.727 | -.012 | .087 |  |  | 11.548 |
|  | Family Support | .021 | .056 | .040 | .383 | .702 | -.089 | .132 | .346 |  |  | 2.892 |
|  | Monthly Income Level | .009 | .045 | .016 | .189 | .850 | -.081 | .098 | .525 |  |  | 1.903 |
|  | History of Depression | .314 | .162 | .358 | 1.933 | .054 | -.006 | .633 | .108 |  |  | 9.294 |
|  | Type of Birth | -.017 | .070 | -.016 | -.237 | .813 | -.154 | .121 | .779 |  |  | 1.284 |
|  | Postpartum Period | -.051 | .045 | -.098 | -1.151 | .251 | -.139 | .037 | .511 |  |  | 1.956 |
|  | #Previous Pregnancy | .026 | .060 | .030 | .439 | .661 | -.091 | .143 | .814 |  |  | 1.228 |
| a. Dependent Variable: Comorbidity of Anxiety and Depressive Symptoms | | | | | | | | | | | | |

*Note*. VIF, Variance Inflation Factors. CI. = Confidence interval

HFT, Health Facility

DVE, Domestic Violence Experience

**Supplemental Table 4** Model with interaction term for PND

|  | | B | S.E. | Wald | df | Sig. | Exp(B) | 95% C.I.for EXP(B) | |
| --- | --- | --- | --- | --- | --- | --- | --- | --- | --- |
|  |  |  |  |  |  |  |  | Lower | Upper |
| Step 1^a^ | MotherAge |  |  | 2.858 | 2 | .239 |  |  |  |
|  | MotherAge(1) | -1.039 | .630 | 2.721 | 1 | .099 | .354 | .103 | 1.216 |
|  | MotherAge(2) | -.599 | .866 | .478 | 1 | .490 | .549 | .101 | 3.003 |
|  | EducLevel |  |  | 4.757 | 3 | .190 |  |  |  |
|  | EducLevel(1) | .700 | .432 | 2.624 | 1 | .105 | 2.013 | .863 | 4.696 |
|  | EducLevel(2) | .097 | .435 | .049 | 1 | .824 | 1.101 | .470 | 2.583 |
|  | EducLevel(3) | -.089 | .417 | .046 | 1 | .831 | .915 | .404 | 2.071 |
|  | HFT(1) | .708 | .412 | 2.945 | 1 | .086 | 2.030 | .904 | 4.555 |
|  | HFT * MotherAge |  |  | 5.651 | 2 | .059 |  |  |  |
|  | HFT(1) by MotherAge(1) | .197 | .728 | .073 | 1 | .787 | 1.217 | .292 | 5.068 |
|  | HFT(1) by MotherAge(2) | 2.829 | 1.194 | 5.614 | 1 | .018 | 16.926 | 1.630 | 175.724 |
|  | Constant | -1.048 | .438 | 5.728 | 1 | .017 | .351 |  |  |
| a. Variable(s) entered on step 1: HFT * Mother Age. | | | | | | | | | |

b. This parameter is set to zero because it is redundant

Nagelkerke = .148; Cox and Snell = .204

**Supplemental Table 5** Model with interaction terms for PNA

|  | | B | S.E. | Wald | df | Sig. | Exp(B) | 95% C.I.for EXP(B) | |
| --- | --- | --- | --- | --- | --- | --- | --- | --- | --- |
|  |  |  |  |  |  |  |  | Lower | Upper |
| Step 1^a^ | MotherAge |  |  | 12.874 | 2 | .002 |  |  |  |
|  | MotherAge(1) | -1.254 | .354 | 12.520 | 1 | .000 | .285 | .143 | .572 |
|  | MotherAge(2) | -.841 | .637 | 1.741 | 1 | .187 | .431 | .124 | 1.504 |
|  | EducLevel |  |  | 9.805 | 3 | .020 |  |  |  |
|  | EducLevel(1) | .169 | .723 | .055 | 1 | .815 | 1.184 | .287 | 4.880 |
|  | EducLevel(2) | 1.814 | .781 | 5.387 | 1 | .020 | 6.134 | 1.326 | 28.371 |
|  | EducLevel(3) | -.312 | .817 | .146 | 1 | .702 | .732 | .148 | 3.630 |
|  | PlaceResid(1) | .140 | .555 | .064 | 1 | .800 | 1.151 | .388 | 3.412 |
|  | IncomeLevel |  |  | 2.928 | 2 | .231 |  |  |  |
|  | IncomeLevel(1) | .881 | .788 | 1.251 | 1 | .263 | 2.414 | .515 | 11.306 |
|  | IncomeLevel(2) | 1.379 | .814 | 2.870 | 1 | .090 | 3.971 | .805 | 19.578 |
|  | HistoryDepression(1) | 1.023 | .549 | 3.479 | 1 | .062 | 2.783 | .949 | 8.157 |
|  | EducLevel * IncomeLevel |  |  | 24.378 | 6 | .000 |  |  |  |
|  | EducLevel(1) by IncomeLevel(1) | 1.486 | 1.173 | 1.604 | 1 | .205 | 4.420 | .443 | 44.055 |
|  | EducLevel(1) by IncomeLevel(2) | 1.433 | 1.133 | 1.599 | 1 | .206 | 4.191 | .455 | 38.613 |
|  | EducLevel(2) by IncomeLevel(1) | -3.730 | 1.228 | 9.220 | 1 | .002 | .024 | .002 | .267 |
|  | EducLevel(2) by IncomeLevel(2) | -2.373 | 1.065 | 4.965 | 1 | .026 | .093 | .012 | .751 |
|  | EducLevel(3) by IncomeLevel(1) | -.259 | 1.049 | .061 | 1 | .805 | .772 | .099 | 6.031 |
|  | EducLevel(3) by IncomeLevel(2) | .656 | 1.090 | .362 | 1 | .547 | 1.927 | .228 | 16.311 |
|  | Constant | -1.561 | .662 | 5.569 | 1 | .018 | .210 |  |  |
| a. Variable(s) entered on step 1: Educ Level * Income Level. | | | | | | | | | |

Nagelkerke = .219; Cox and Snell = .304

**Supplemental Table 6** BLR Model with interaction terms for comorbidity of anxiety and depression

|  | | B | S.E. | Wald | df | Sig. | Exp(B) | 95% C.I.for EXP(B) | |
| --- | --- | --- | --- | --- | --- | --- | --- | --- | --- |
|  |  |  |  |  |  |  |  | Lower | Upper |
| Step 1^a^ | MotherAge |  |  | 6.035 | 2 | .049 |  |  |  |
|  | MotherAge(1) | -.872 | .850 | 1.053 | 1 | .305 | .418 | .079 | 2.211 |
|  | MotherAge(2) | 1.900 | 1.011 | 3.533 | 1 | .060 | 6.683 | .922 | 48.444 |
|  | EducLevel |  |  | 3.875 | 3 | .275 |  |  |  |
|  | EducLevel(1) | .924 | .920 | 1.009 | 1 | .315 | 2.520 | .415 | 15.284 |
|  | EducLevel(2) | 1.867 | 1.059 | 3.109 | 1 | .078 | 6.469 | .812 | 51.538 |
|  | EducLevel(3) | .415 | 1.052 | .155 | 1 | .693 | 1.514 | .193 | 11.911 |
|  | PlaceResid(1) | -.773 | .566 | 1.867 | 1 | .172 | .461 | .152 | 1.399 |
|  | IncomeLevel |  |  | 2.930 | 2 | .231 |  |  |  |
|  | IncomeLevel(1) | 1.378 | .934 | 2.179 | 1 | .140 | 3.968 | .637 | 24.738 |
|  | IncomeLevel(2) | -.085 | .979 | .008 | 1 | .931 | .919 | .135 | 6.260 |
|  | HistoryDepression(1) | 1.270 | .552 | 5.299 | 1 | .021 | 3.560 | 1.208 | 10.495 |
|  | HFT(1) | 1.237 | .465 | 7.092 | 1 | .008 | 3.446 | 1.386 | 8.565 |
|  | EducLevel * MotherAge |  |  | 8.578 | 6 | .199 |  |  |  |
|  | EducLevel(1) by MotherAge(1) | 1.616 | 1.159 | 1.944 | 1 | .163 | 5.035 | .519 | 48.837 |
|  | EducLevel(1) by MotherAge(2) | -22.716 | 22058.853 | .000 | 1 | .999 | .000 | .000 | . |
|  | EducLevel(2) by MotherAge(1) | -.670 | 1.139 | .346 | 1 | .557 | .512 | .055 | 4.775 |
|  | EducLevel(2) by MotherAge(2) | -2.342 | 1.611 | 2.113 | 1 | .146 | .096 | .004 | 2.261 |
|  | EducLevel(3) by MotherAge(1) | -.942 | 1.120 | .707 | 1 | .400 | .390 | .043 | 3.504 |
|  | EducLevel(3) by MotherAge(2) | -.145 | 1.361 | .011 | 1 | .915 | .865 | .060 | 12.460 |
|  | EducLevel * IncomeLevel |  |  | 12.799 | 6 | .046 |  |  |  |
|  | EducLevel(1) by IncomeLevel(1) | .334 | 1.282 | .068 | 1 | .794 | 1.397 | .113 | 17.228 |
|  | EducLevel(1) by IncomeLevel(2) | .251 | 1.251 | .040 | 1 | .841 | 1.285 | .111 | 14.910 |
|  | EducLevel(2) by IncomeLevel(1) | -2.785 | 1.289 | 4.673 | 1 | .031 | .062 | .005 | .771 |
|  | EducLevel(2) by IncomeLevel(2) | -.634 | 1.277 | .247 | 1 | .619 | .530 | .043 | 6.479 |
|  | EducLevel(3) by IncomeLevel(1) | -1.157 | 1.211 | .912 | 1 | .340 | .314 | .029 | 3.379 |
|  | EducLevel(3) by IncomeLevel(2) | 1.473 | 1.248 | 1.391 | 1 | .238 | 4.360 | .377 | 50.372 |
|  | Constant | -2.861 | .921 | 9.649 | 1 | .002 | .057 |  |  |
| a. Variable(s) entered on step 1: EducLevel * MotherAge , EducLevel * IncomeLevel . | | | | | | | | | |

**Supplemental Table 7** MLR Model with interaction terms for comorbidity of anxiety and depression

| ADComorbid^a^ | | B | Std. Error | Wald | df | Sig. | Exp(B) | 95% Confidence Interval for Exp(B) | |
| --- | --- | --- | --- | --- | --- | --- | --- | --- | --- |
|  |  |  |  |  |  |  |  | Lower Bound | Upper Bound |
| Anxiety | Intercept | 2.023 | 1.087 | 3.461 | 1 | .063 |  |  |  |
|  | [EducLevel=1] * [HFT=1] | -.007 | .861 | .000 | 1 | .993 | .993 | .184 | 5.372 |
|  | [EducLevel=1] * [HFT=2] | 1.684 | 1.151 | 2.141 | 1 | .143 | 5.389 | .565 | 51.430 |
|  | [EducLevel=2] * [HFT=1] | -.079 | 1.016 | .006 | 1 | .938 | .924 | .126 | 6.766 |
|  | [EducLevel=2] * [HFT=2] | 2.091 | 1.164 | 3.225 | 1 | .073 | 8.091 | .826 | 79.263 |
|  | [EducLevel=3] * [HFT=1] | -.778 | .862 | .815 | 1 | .367 | .459 | .085 | 2.486 |
|  | [EducLevel=3] * [HFT=2] | 2.269 | 1.136 | 3.992 | 1 | .046 | 9.671 | 1.044 | 89.586 |
|  | [EducLevel=4] * [HFT=1] | -.103 | .790 | .017 | 1 | .897 | .902 | .192 | 4.246 |
|  | [EducLevel=4] * [HFT=2] | 0^b^ | . | . | 0 | . | . | . | . |
|  | [IncomeLevel=1] * HistoryDepression | -.955 | .589 | 2.628 | 1 | .105 | .385 | .121 | 1.221 |
|  | [IncomeLevel=2] * HistoryDepression | -.737 | .558 | 1.747 | 1 | .186 | .479 | .160 | 1.428 |
|  | [IncomeLevel=3] * HistoryDepression | -.593 | .610 | .945 | 1 | .331 | .553 | .167 | 1.826 |
| Depression | Intercept | 3.268 | 1.057 | 9.565 | 1 | .002 |  |  |  |
|  | [EducLevel=1] * [HFT=1] | -.452 | .857 | .278 | 1 | .598 | .637 | .119 | 3.414 |
|  | [EducLevel=1] * [HFT=2] | 1.459 | 1.136 | 1.648 | 1 | .199 | 4.300 | .464 | 39.866 |
|  | [EducLevel=2] * [HFT=1] | -1.217 | 1.131 | 1.158 | 1 | .282 | .296 | .032 | 2.716 |
|  | [EducLevel=2] * [HFT=2] | 1.449 | 1.161 | 1.558 | 1 | .212 | 4.260 | .438 | 41.476 |
|  | [EducLevel=3] * [HFT=1] | -.146 | .769 | .036 | 1 | .850 | .864 | .191 | 3.906 |
|  | [EducLevel=3] * [HFT=2] | 1.221 | 1.148 | 1.131 | 1 | .288 | 3.391 | .357 | 32.186 |
|  | [EducLevel=4] * [HFT=1] | -.344 | .765 | .202 | 1 | .653 | .709 | .158 | 3.177 |
|  | [EducLevel=4] * [HFT=2] | 0^b^ | . | . | 0 | . | . | . | . |
|  | [IncomeLevel=1] * HistoryDepression | -1.427 | .580 | 6.056 | 1 | .014 | .240 | .077 | .748 |
|  | [IncomeLevel=2] * HistoryDepression | -1.282 | .547 | 5.487 | 1 | .019 | .277 | .095 | .811 |
|  | [IncomeLevel=3] * HistoryDepression | -1.195 | .602 | 3.944 | 1 | .047 | .303 | .093 | .984 |
| Anxious-Depressed | Intercept | 2.133 | 1.107 | 3.711 | 1 | .054 |  |  |  |
|  | [EducLevel=1] * [HFT=1] | -.720 | .954 | .570 | 1 | .450 | .487 | .075 | 3.157 |
|  | [EducLevel=1] * [HFT=2] | 1.620 | 1.151 | 1.981 | 1 | .159 | 5.051 | .529 | 48.192 |
|  | [EducLevel=2] * [HFT=1] | .115 | .971 | .014 | 1 | .906 | 1.122 | .167 | 7.530 |
|  | [EducLevel=2] * [HFT=2] | 2.324 | 1.165 | 3.982 | 1 | .046 | 10.216 | 1.042 | 100.139 |
|  | [EducLevel=3] * [HFT=1] | -1.136 | .887 | 1.640 | 1 | .200 | .321 | .056 | 1.827 |
|  | [EducLevel=3] * [HFT=2] | 1.691 | 1.151 | 2.160 | 1 | .142 | 5.426 | .569 | 51.756 |
|  | [EducLevel=4] * [HFT=1] | -1.282 | .920 | 1.939 | 1 | .164 | .278 | .046 | 1.686 |
|  | [EducLevel=4] * [HFT=2] | 0^b^ | . | . | 0 | . | . | . | . |
|  | [IncomeLevel=1] * HistoryDepression | -1.101 | .606 | 3.296 | 1 | .069 | .333 | .101 | 1.092 |
|  | [IncomeLevel=2] * HistoryDepression | -.615 | .568 | 1.172 | 1 | .279 | .541 | .177 | 1.647 |
|  | [IncomeLevel=3] * HistoryDepression | -.733 | .637 | 1.325 | 1 | .250 | .480 | .138 | 1.674 |

a. The reference category is: No Symptoms.

b. This parameter is set to zero because it is redundant.

**Table 6** Table showing multicollinearity (Variance Inflation Factors)

| **Coefficients^a^** | | | | | | | | | |
| --- | --- | --- | --- | --- | --- | --- | --- | --- | --- |
| Model | Unstandardized  Coefficients | | Unstandardized  Coefficients | t | Sig. | 95.0% confidence interval for B | | Collinearity  Statistics | |
|  | B | Std. Error | Beta |  |  | Lower bound | Upper bound | Tolerance | VIF |
| (Constant) | 10.272 | 1.662 |  | 6.181 | .000 | 6.999 | 13.545 |  |  |
| Age | -.288 | .379 | -.046 | -.759 | .449 | -1.035 | .459 | .911 | 1.098 |
| Level of Education | -.563 | .215 | -.155 | -2.621 | .009 | -.986 | -.140 | .971 | 1.030 |
| Health Facility Type | 1.990 | .549 | .232 | 3.623 | .000 | .908 | 3.072 | .826 | 1.210 |
| Place of Residence | 1.548 | .935 | .188 | 1.656 | .099 | -.293 | 3.390 | .263 | 3.800 |
| DVE | -4.209 | 1.607 | -.519 | -2.620 | .009 | -7.373 | -1.045 | .087 | 11.548 |
| Family Support | .425 | .496 | .085 | .856 | .393 | -.552 | 1.401 | .346 | 2.892 |
| Monthly Income level | .243 | .402 | .049 | .606 | .545 | -.548 | 1.034 | .525 | 1.903 |
| History of Depression | 1.898 | 1.436 | .235 | 1.322 | .187 | -.930 | 4.726 | .108 | 9.294 |
| Type of Birth | .252 | .618 | .027 | .407 | .684 | -.966 | 1.469 | .779 | 1.284 |
| # Previous Preg. | .659 | .527 | .081 | 1.250 | .212 | -.379 | 1.698 | .814 | 1.228 |
| Postpartum Period | -.631 | .395 | -.130 | -1.597 | .111 | -1.409 | .147 | .511 | 1.956 |

a. Dependent variable: postnatal depression (EPDS Score ≥ 13)

**Appendix 2**

**Table 7** Table showing multicollinearity (Variance Inflation Factors)

| **Coefficients^a^** | | | | | | | | | |
| --- | --- | --- | --- | --- | --- | --- | --- | --- | --- |
| Model | Unstandardized  Coefficients | | Unstandardized  Coefficients | t | Sig. | 95.0% confidence interval for B | | Collinearity  Statistics | |
|  | B | Std. Error | Beta |  |  | Lower bound | Upper bound | Tolerance | VIF |
| (Constant) | 5.516 | 1.507 | -.234 | 3.662 | .000 | 2.550 | 8.483 |  |  |
| Age | -1.357 | .344 | -.130 | -3.945 | .000 | -2.034 | -.680 | .911 | 1.098 |
| Level of Education | -.440 | .195 | .120 | -2.259 | .025 | -.823 | -.056 | .971 | 1.030 |
| Health Facility Type | .958 | .498 | .197 | 1.924 | .055 | -.023 | 1.939 | .826 | 1.210 |
| Place of Residence | 1.512 | .847 | -.090 | 1.784 | .076 | -.157 | 3.181 | .263 | 3.800 |
| DVE | -.685 | 1.457 | .160 | -.470 | .639 | -3.553 | 2.184 | .087 | 11.548 |
| Family Support | .745 | .450 | .158 | 1.658 | .099 | -.140 | 1.631 | .346 | 2.892 |
| Monthly Income level | .737 | .364 | .108 | 2.024 | .044 | .020 | 1.454 | .525 | 1.903 |
| History of Depression | .816 | 1.302 | -.086 | .627 | .531 | -1.747 | 3.380 | .108 | 9.294 |
| Type of Birth | -.748 | .560 | .036 | -1.334 | .183 | -1.851 | .356 | .779 | 1.284 |
| # Previous Preg. | .278 | .478 | -.257 | .581 | .562 | -.663 | 1.219 | .814 | 1.228 |
| Postpartum Period | -1.164 | .358 | -.234 | -3.250 | .001 | -1.869 | -.459 | .511 | 1.956 |

a. Dependent variable: postnatal anxiety (HADS-A Score +8)

**Appendix 3**

**Table 8** Model with interaction term

|  | | B | S.E. | Wald | df | Sig. | Exp(B) | 95% C.I.for EXP(B) | |
| --- | --- | --- | --- | --- | --- | --- | --- | --- | --- |
|  |  |  |  |  |  |  |  | Lower | Upper |
| Step 1^a^ | MotherAge |  |  | 2.858 | 2 | .239 |  |  |  |
|  | MotherAge(1) | -1.039 | .630 | 2.721 | 1 | .099 | .354 | .103 | 1.216 |
|  | MotherAge(2) | -.599 | .866 | .478 | 1 | .490 | .549 | .101 | 3.003 |
|  | EducLevel |  |  | 4.757 | 3 | .190 |  |  |  |
|  | EducLevel(1) | .700 | .432 | 2.624 | 1 | .105 | 2.013 | .863 | 4.696 |
|  | EducLevel(2) | .097 | .435 | .049 | 1 | .824 | 1.101 | .470 | 2.583 |
|  | EducLevel(3) | -.089 | .417 | .046 | 1 | .831 | .915 | .404 | 2.071 |
|  | HFT(1) | .708 | .412 | 2.945 | 1 | .086 | 2.030 | .904 | 4.555 |
|  | HFT * MotherAge |  |  | 5.651 | 2 | .059 |  |  |  |
|  | HFT(1) by MotherAge(1) | .197 | .728 | .073 | 1 | .787 | 1.217 | .292 | 5.068 |
|  | HFT(1) by MotherAge(2) | 2.829 | 1.194 | 5.614 | 1 | .018 | 16.926 | 1.630 | 175.724 |
|  | Constant | -1.048 | .438 | 5.728 | 1 | .017 | .351 |  |  |
| a. Variable(s) entered on step 1: HFT * Mother Age. | | | | | | | | | |

b. This parameter is set to zero because it is redundant

Nagelkerke = .148; Cox and Snell = .204

**Appendix 4**

**Table 9** Model with interaction term

|  | | B | S.E. | Wald | df | Sig. | Exp(B) | 95% C.I.for EXP(B) | |
| --- | --- | --- | --- | --- | --- | --- | --- | --- | --- |
|  |  |  |  |  |  |  |  | Lower | Upper |
| Step 1^a^ | MotherAge |  |  | 12.874 | 2 | .002 |  |  |  |
|  | MotherAge(1) | -1.254 | .354 | 12.520 | 1 | .000 | .285 | .143 | .572 |
|  | MotherAge(2) | -.841 | .637 | 1.741 | 1 | .187 | .431 | .124 | 1.504 |
|  | EducLevel |  |  | 9.805 | 3 | .020 |  |  |  |
|  | EducLevel(1) | .169 | .723 | .055 | 1 | .815 | 1.184 | .287 | 4.880 |
|  | EducLevel(2) | 1.814 | .781 | 5.387 | 1 | .020 | 6.134 | 1.326 | 28.371 |
|  | EducLevel(3) | -.312 | .817 | .146 | 1 | .702 | .732 | .148 | 3.630 |
|  | PlaceResid(1) | .140 | .555 | .064 | 1 | .800 | 1.151 | .388 | 3.412 |
|  | IncomeLevel |  |  | 2.928 | 2 | .231 |  |  |  |
|  | IncomeLevel(1) | .881 | .788 | 1.251 | 1 | .263 | 2.414 | .515 | 11.306 |
|  | IncomeLevel(2) | 1.379 | .814 | 2.870 | 1 | .090 | 3.971 | .805 | 19.578 |
|  | HistoryDepression(1) | 1.023 | .549 | 3.479 | 1 | .062 | 2.783 | .949 | 8.157 |
|  | EducLevel * IncomeLevel |  |  | 24.378 | 6 | .000 |  |  |  |
|  | EducLevel(1) by IncomeLevel(1) | 1.486 | 1.173 | 1.604 | 1 | .205 | 4.420 | .443 | 44.055 |
|  | EducLevel(1) by IncomeLevel(2) | 1.433 | 1.133 | 1.599 | 1 | .206 | 4.191 | .455 | 38.613 |
|  | EducLevel(2) by IncomeLevel(1) | -3.730 | 1.228 | 9.220 | 1 | .002 | .024 | .002 | .267 |
|  | EducLevel(2) by IncomeLevel(2) | -2.373 | 1.065 | 4.965 | 1 | .026 | .093 | .012 | .751 |
|  | EducLevel(3) by IncomeLevel(1) | -.259 | 1.049 | .061 | 1 | .805 | .772 | .099 | 6.031 |
|  | EducLevel(3) by IncomeLevel(2) | .656 | 1.090 | .362 | 1 | .547 | 1.927 | .228 | 16.311 |
|  | Constant | -1.561 | .662 | 5.569 | 1 | .018 | .210 |  |  |
| a. Variable(s) entered on step 1: Educ Level * Income Level. | | | | | | | | | |

Nagelkerke = .219; Cox and Snell = .304
